# “I Had To Be Brave”: Unveiling the Remarkable Resilience of Children Who Experienced Sexual Abuse

**DOI:** 10.1007/s40653-025-00714-0

**Published:** 2025-05-07

**Authors:** Chantelle van der Walt, Daphney Mawila-Chauke

**Affiliations:** Faculty of Education, University of Johannesburg, GNA 226, Robert Sobukwe Building Soweto Campus, Johannesburg, 1809 South Africa

**Keywords:** Contextual factors, Individual factors, Relational factors, Resilience, Sexual abuse, Social-ecological framework

## Abstract

Sexual abuse is a global problem with profound consequences for the well-being of children. It often results in mental health disorders such as depression, post-traumatic stress disorder (PTSD) and anxiety. It can lead to social withdrawal, substance use and abuse, aggression and suicide. Despite the adversities faced by children who have experienced sexual abuse, some are resilient and do not succumb to the subsequent consequences. Therefore, this South African study aimed to explore the protective factors that enable the resilience of children who have been sexually abused. In line with the qualitative research, a phenomenological research design was used. Six female children in two children’s homes were purposefully chosen for this study. Ungar’s theory of resilience was the theoretical framework that underpinned this study. Data was collected through semi-structured interviews and analysed using Braun and Clarke’s (Braun, V., & Clarke, V. (2006). Qualitative Research in Psychology, 3(2):77–101) six-step thematic approach. The findings, based on participants’ verbatim responses, identified individual, relational, and contextual factors as essential social-ecological resources that support resilience in children who have experienced sexual abuse. Individual factors included qualities such as a capacity to help others, personal interests, bravery, and a forward-looking vision. Relational factors involved supportive social connections and caregiver support, while contextual factors included role models, religion and spirituality, and support from teachers. These findings highlight the importance of stakeholders in children’s social ecology in developing and implementing supportive measures.

## Introduction

Sexual abuse is a pervasive global issue with profound implications for children’s well-being. Masilo and Davhana-Naselesele (2017) (stated that over 40% of children worldwide had experienced sexual abuse. South Africa holds the fourth-highest sexual abuse rate globally (World Health Organization [WHO], 2022). In a national survey, Richter et al. (2018) reported that 9,730 young people between the ages of 15 and 17 in South Africa reported that they had experienced sexual abuse. In addition, the South African Institute for Security Studies (2010) revealed that 20,141 sexual crimes were committed against children from 2008 to 2009, with a significant portion recorded against children under the age of 15. The Health-E News (Gwala, 2021) also specified that one-third of girls in South Africa had experienced sexual abuse, and approximately one in every five children would have been sexually assaulted. Furthermore, Haslam et al. (2021) explain that in South Africa, approximately 50,000 incidents of sexual abuse were reported to law enforcement authorities between 2016 and 2017. This approximation translates to an average of 136 reported incidents of sexual abuse per day (Haslam et al., 2021). As of the fiscal year 2022/2023, nearly 53,900 South Africans reported that they had experienced a sexual crime (Ajayi et al., 2021). Of these reports, more than 80% were cases of rape, with almost 7,600 individuals disclosing incidents of sexual assault. The above-mentioned prevalence of sexual abuse in South Africa shows that children are at risk and calls for measures to be put in place to protect them.

Sexual abuse is one of the forms of child abuse and maltreatment that is experienced by children. The World Health Organization (WHO, 2010, p.16) defines sexual abuse as “the involvement of a child in sexual activity that he or she does not fully comprehend and is unable to give informed consent to, or for which the child is not developmentally prepared, or else that violates the laws or social taboos of society”. Sexual abuse encompasses inappropriate touching, forced oral and genital penetration, sexual harassment exerted mostly by individuals in positions of authority, and involvement in pornography (WHO, 2010). Richter et al. (2018) assert that sexual abuse occurs in instances where there is a lack of consent to sexual interaction or contact, particularly when the child is below the age of 18.

Sexual abuse is detrimental to the development of children and their overall well-being. The aftermath of sexual abuse often includes mental health challenges and various psychopathologies such as depression, post-traumatic stress disorder (PTSD), anxiety and personality disorders (Kratzer et al., 2020; Richter et al., 2018; Sedlak et al., 2010). Kratzer et al. (2020) added that sexual abuse can hinder social growth, leading to psychosocial problems and difficulties in interpersonal relationships. Sexual abuse also has profound physical health implications, including medically unexplained symptoms and increased risk of sexually transmitted diseases (STDs) (Heger et al., 2002). Santaularia et al. (2014) showed a connection between chronic illnesses and experiences of sexual abuse. This suggests that survivors of such abuse may develop long-term health issues. It highlights the importance of effective interventions and psychological support, as individual coping mechanisms can influence health outcomes positively (Runyan et al., 2002). Academic performance may also be affected by sexual abuse (Narad & Abdullah, 2016; Osiki, 2001). Sexual abuse has been associated with detrimental effects, including lower psychometric test scores, increased school absenteeism, and emotional distress, negatively impacting academic performance (Buckle et al., 2005; Hlupo & Tsikira, 2012). Therefore, the effects of sexual abuse on academic and professional prospects are enduring (Altafim & Linhares, 2016). As noted above, sexual abuse has dire consequences on the individuals’ functioning, thus, the need for protective factors is highlighted.

Protective factors are associated with a lower probability of unfavourable outcomes and serve to alleviate the impact of risk factors. These factors can be seen as positive elements that counterbalance adverse circumstances (Claussen et al., 2013). Further, the Substance Abuse and Mental Health Services Administration (SAMHSA, 2023) describe protective factors as the conditions or attributes that foster mental well-being and equip individuals with the resilience necessary to navigate life’s challenges successfully. Protective factors play a critical role in safeguarding children from sexual abuse and reducing future victimisation. Joshi (2018) asserts that protective factors such as a supportive environment, characterised by stable neighbourhoods and community cohesion, play a crucial role in protecting children from abuse. Communities with strong social networks and resources are more resilient in the face of adversities (Joshi, 2018; Seth, 2013). Within the familial context, a protective mother, positive parent-child relationships, and the presence of multiple caregivers can significantly mitigate vulnerability (Berliner, 2011; Fergusson et al., 1996; Finkelhor et al., 2010). Casteix (2018) suggests that building children’s self-confidence and providing them with emotional support enhances their resilience and reduces susceptibility to exploitation. Moreover, educating children on the safe use of technology and social media, while promoting digital literacy, empowers them to protect themselves from online predators (Casteix, 2018; Killean et al., 2022). It is thus clear that protective factors are crucial to enable children’s resilience.

Ungar (2008) defines resilience as “both the capacity of individuals to navigate their way to the psychological, social, cultural, and physical resources that sustain their well-being, and their capacity individually and collectively to negotiate for these resources to be provided and experienced in culturally meaningful ways (p. 225).” This view of resilience is ideal for this South African study where interdependence is valued, and resilience should be considered from a multiple perspective. The concept of “Ubuntu” in the African worldview represents the process of becoming an ethical human being (Makgahlela & Sodi, 2017). According to Archbishop Tutu (2006), a person with *Ubuntu* embodies the idea that “I am because you are.” Tutu describes this perspective by saying, “Ubuntu is not, ‘I think, therefore I am.’ It says rather: ‘I am human because I belong. I participate. I share.‘” This philosophy emphasises the interconnectedness of individuals within a community and the essence of humanity as a shared experience. Shumba (2011) describes “Ubuntu” as an ethical and moral framework that promotes sustainable living by emphasising collective responsibility and shared agency. These values are fundamental to behaviour management and character development. This African belief in interconnectedness reflects the broader perspective of resilience as well, which emphasises community, mutual support, and collective responsibility in fostering well-being (Theron & Phasha, 2015; Theron, 2010; van Breda, 2018).

Ubuntu asserts that resilience is not merely an individual trait, but a communal process nurtured through shared values of care and interconnectedness (Lefa, 2015; Letseka, 2014). This idea aligns closely with Ungar’s (2013) social-ecological theory, which highlights the importance of individual, relational, and contextual factors in overcoming adversity. According to Ungar (2015), resilience is embedded within a social ecology where family, social structures, services (e.g., welfare, healthcare, and education), and cultural values collaboratively support positive adjustment, as also noted by Bottrell (2009). Ungar further emphasises that resilience relies not only on individual agency but equally on the supportive capacities of one’s social and physical environment to foster positive development under stress. Van Rensburg et al. (2019) explain resilience as a process that requires individuals to work together with their surrounding social environments to achieve positive outcomes. Social-ecological factors such as caregiver support, spirituality, and social connections have been noted by Afifi et al. (2016) as critical in fostering resilience, enabling individuals to confront and navigate challenges effectively. In this study, the Ungar’s holistic framework assisted in understanding the individual and contextual factors that enable the resilience of children who have been sexually abused.

Research in the field of resilience is evolving across the globe. SAMHSA (2023) states that while risk factors render children vulnerable to adverse outcomes, protective factors counterbalance adverse circumstances, fostering children’s mental well-being and resilience. Various studies have been undertaken on the resilience of individuals with adversities across the globe. Babu et al. (2021) identified a range of predictive factors that may enhance or diminish an individual’s resilience in the face of sexual abuse. Such factors include the individual’s emotional, attitudinal, and belief-related characteristics, the circumstances surrounding the abuse (such as the perpetrator’s identity or the age at which abuse began), the quality of interpersonal relationships and both the immediate environment and the broader social and environmental contexts (Theron & Theron, 2010). Therefore, understanding the complex interplay between individual characteristics and environmental factors is critical in identifying factors that may enhance or diminish an individual’s resilience in the face of sexual abuse. The goal of this study was to explore the factors that enable resilience of children who have been sexually abused. Despite the well-documented global impact of sexual abuse (Jenny & Crawford-Jakubiak, 2013), the resilience of those who have experienced such trauma remains underexplored, particularly within a social-ecological context. In addition, very few studies have been conducted on the resilience of children who have been sexually abused, specifically in South Africa. Thus, this study aimed to close this gap in literature. It also aimed to provide insights that may be used to develop resilience-building programmes to support children who have experienced sexual abuse. This study also makes a novel contribution to research by using a qualitative approach to allow participants to voice first-hand accounts of their lived experiences of their resilience despite sexual abuse, especially in South Africa.

## Methodology

### Procedure

This study adopted a qualitative research approach to investigate participants’ resilience. This approach enables researchers to understand individuals’ experiences in their social contexts (Aspers & Corte, 2019; Meyers, 2009) and empowers the participants. It facilitated an in-depth exploration of the protective factors that enable the resilience of children who have been sexually abused, from their own perspectives and within their social environments. A phenomenological research design was employed to delve into the meanings individuals ascribe to their experiences (Creswell, 2013). This design aligned with the study’s aim of exploring factors that enable resilience in children based on their lived experiences of sexual abuse.

Participants were purposefully selected from two children’s homes in Gauteng, South Africa—one in Pretoria and the other in Johannesburg. These homes serve as protective environments for children who have experienced neglect, abuse, orphanhood, and other difficulties, including sexual abuse. The selection process was guided by social workers at the children’s homes, who first identified children with a history of sexual abuse who had been observed by social workers to be coping well. They had adjusted positively at the children’s home and at school according to the school records. As this study focused on vulnerable children, social workers only recommended children who showed resilience and had successfully completed the therapeutic intervention processes for the study. This study was therefore critical as it explored the protective factors that enabled the participating children to thrive, despite exposure to adversity, while others struggle.

The participants in this study were exclusively female, as the selected children’s homes only accommodated girls. The sample consisted of two Afrikaans-speaking white children and four black English-speaking children, all aged between 14 and 17 years. Interviews were conducted in English in which language all participants were able to converse comfortably. No remuneration was provided for participation in the study. The authors of this study did not have a relationship with the participants before undertaking this study; thus, there was no potential influence on the findings in this regard.

### Data Collection

Semi-structured interviews were used to collect data. The interviews were conducted in a quiet boardroom, free from distractions. Participants were not asked questions directly related to the sexual abuse. This was to decrease the risk of re-traumatising them. Despite this precaution, and to mitigate any risk of potential re-traumatisation, a qualified psychologist was present during the interview sessions to assist with immediate psychological intervention if necessary. The psychologist was available during and after data collection because of the vulnerability of the participants, who had also given their consent for the psychologist to be present and to provide additional intervention should it be necessary. Each interview lasted thirty minutes, and was conducted in English, the language in which all participants were fluent (Dejonckheere & Vaughn, 2021).

The study aimed to explore the overarching research question: “What factors enable the resilience of children who have experienced sexual abuse?” To gain deeper insight into the participants’ resilience factors, semi-structured interviews were conducted. The interview questions were formulated to understand resilience at the individual level, with peers, with caregiver support, and within the broader context of the children’s environment. For example, under the individual factors, participants were asked to reflect on their personal qualities, strengths, and experiences in facing challenges, such as: “How do you handle it when things don’t go as planned or when you make a mistake?” and “Can you think of a time when you had to be brave and face a fear?” This allowed participants to express the way they drew on personal resources to navigate adversity. In the peer support section, questions like “Can you tell me about a time when someone else supported you when you needed it?” aimed to uncover how relationships with friends and peers contribute to their resilience. Similarly, under caregiver support, participants were asked, “What are some things that your caregiver does to help you feel safe and loved?” to explore the role of caregivers in fostering a sense of security and emotional stability. Lastly, questions in the contextual factors, such as “What activities do you do in your community, at home, or at school that make you feel happy?” helped to reveal the broader contextual and environmental factors that support resilience, including community and spiritual involvement. By structuring the interviews around these themes, the study provided a comprehensive understanding of the resilience of children who have experienced sexual abuse. This multi-layered exploration, rooted in Ungar’s social-ecological theory, offered unique insights into the resilience-building processes of these vulnerable children.

### Data Analysis

In this study, thematic data analysis was used to analyse collected data. Thematic data analysis offers qualitative researchers a systematic approach to uncover recurring patterns and themes within collected data (Wong, 2008). It involves several key steps to systematically explore and organise the data, providing valuable insights into the phenomenon under study (Wong, 2008). Braun and Clarke’s (2006) six steps of thematic data analysis were used to guide researchers, from the first step of familiarising themselves with the data to producing a comprehensive report at the end of the process.

#### Step 1: Familiarisation with Data

Immersion in the data involves actively engaging with interview transcripts and observation notes.

#### Step 2: Generating Initial Codes

Initial coding is the process of identifying similarities in the data (Braun & Clarke, 2006). We used line-by-line inductive coding to group similar ideas that emerged from the data. This step involves extracting words, phrases, and sentences that represent key themes and concepts present in the data.

#### Step 3: Searching for Themes

Codes are organised into thematic piles, allowing for the identification of overarching themes and subthemes that emerge from the data.

#### Step 4: Theme Review

In Phase 4, the themes are reviewed to ensure they make sense and fit together logically. Any issues or inconsistencies are dealt with. This involves carefully checking each theme for problems or contradictions. If a theme does not match the data or fit with the others, it is either adjusted to make it fit better or removed entirely. The aim is to create themes that accurately reflect the data.

#### Step 5: Defining and Naming Themes

Each theme is clearly defined and named to accurately summarise and represent the content. The subthemes in this study were derived directly from the participants’ own language and experiences, allowing the themes to authentically reflect their lived realities. This approach ensured that the analysis stayed closely aligned with the participants’ voices, capturing the nuances of how they perceived and navigated their experiences of resilience. The researchers ensured that the themes accurately represented the children’s perspectives by allowing the data to guide their development. While emergent themes played a key role, priori categories had already been established to frame the interview questions. By integrating both predefined categories and themes that emerged from the data, this method provided a deeper understanding of resilience as it manifested in the specific context of children’s homes for those who had experienced sexual abuse.

#### Step 6: Producing the Report

The final phase involves synthesising the findings into a comprehensive report that lists the identified themes and subthemes.

To ensure qualitative rigour in this study, several key steps were implemented. They were aligned with the criteria outlined by Gunawan (2015), namely, credibility, dependability, transferability, and confirmability. Credibility was prioritised through member checks and validation processes, which involved revisiting the children’s homes to verify the accuracy of the findings with the participants, thereby ensuring that their experiences were authentically represented in the study. The inclusion of diverse perspectives from children across two different homes further enhanced the credibility of the results by providing a more comprehensive understanding of the topic (Leedy et al., 2019). Dependability was maintained by meticulously documenting the research methodology and analysis procedures, following rigorous criteria for research evaluation and emphasising thoroughness and consistency in data collection and analysis. This clear explanation of processes aimed to enhance the reliability and overall quality of the findings (Gunawan, 2015). Transferability was addressed through semi-structured interviews that gathered rich insights into individual experiences related to the topic.

While recognising that qualitative findings are not always directly transferable to other contexts, the inclusion of diverse perspectives allowed for a more nuanced understanding of resilience among children who had experienced sexual abuse in children’s homes in Johannesburg and Pretoria, enriching the relevance of the study’s findings to other settings. Lastly, confirmability was ensured through collaboration with a co-author, who played a crucial role in reviewing and refining the data interpretations to maintain the integrity of the research, ensuring that the conclusions drawn were grounded in the participants’ actual experiences rather than the researcher’s personal biases or imagination (Anney, 2014). Collectively, these steps fostered a rigorous qualitative research process, enhancing the trustworthiness and reliability of the study’s findings.

### Ethical Considerations

Interviews took place individually at the different children’s homes in a quiet boardroom, with prior ethical clearance granted by the XXXX Ethics Committee (Reference number: XXXXX). Relevant documents were sent to the management of the two children’s homes in Johannesburg and Pretoria and approval was granted for the study. Informed consent was obtained from caregivers in writing for participants under 16 years old. The consent process involved a detailed explanation of the study’s purpose and an opportunity for participants and their case managers/guardians to ask questions. All participants were required to sign assent forms as their consent to be part of the study.

Interviews were conducted one-on-one, which excluded the possibility of the participants knowing each other’s situations or who had participated in the study. Additionally, there was no relationship between the participants and the authors, other than the relationship established during the interview process.

Participants were informed that their involvement in the study was entirely voluntary, and they had the right to withdraw at any time without facing any negative consequences. Confidentiality and anonymity were strictly maintained, with pseudonyms used throughout the study and all data securely stored in either a private, locked cabinet or a password-protected file accessible only to the researchers. Interviews took place in the presence of a registered educational psychologist, who followed up with participants one week later to ensure no distress had resulted from participation. No distress was reported, observed, or identified. Importantly, the participants were fully informed about all aspects of the study and signed permission was obtained from all of them before commencing the study.

## Results

The demographic table of participants (Table 1) provides a concise overview of each participant.

**Table 1:** Demographic overview

Sex	Age	Race
Female	14 years old	Black
Female	14 years old	Black
Female	15 years old	Black
Female	15 years old	White
Female	16 years old	Black
Female	17 years old	White

Data analysis used a combined approach, starting with a priori coding, where existing frameworks guided the initial categorisation of data. Data analysis yielded three main themes, namely: (1) individual factors (2) relational factors and (3) contextual factors. These themes, detailed in Table 2, are identified as key components of resilience for children who have experienced sexual abuse. This was followed by an inductive coding that allowed additional insights to emerge organically, leading to the identification of subthemes. Together with their subthemes, these themes highlight the various factors that support participants’ ability to cope with challenges and demonstrate resilience. This section provides an in-depth exploration of each theme and subtheme, interwoven with participants’ verbatim responses to capture their perspectives more fully.

The following table illustrates the key themes and their corresponding subthemes.

**Table 2:** Resilience-enabling themes and subthemes

Themes	Subthemes
Individual factors	Ability to help others Interests Bravery Vision
Relational factors	Supportive social connections Caregivers’ support Teamwork and collaboration
Contextual factors	Role models Religion and spirituality Support from schoolteachers

### Individual Factors

Participants reported that individual factors enabled their resilience. These factors were individual traits, strengths, and strategies individuals use to deal with adversities. Within the main theme of individual factors, four subthemes were generated, namely, 1) Ability to help others 2.) Interests 3) Bravery, and 4) Vision.

#### Ability to help others

Participants emphasised that their ability to help friends contributed significantly to their resilience. Supporting others during difficult times demonstrates their empathy and compassion. It also facilitates personal healing and serves as a source of strength and fulfilment by fostering a sense of purpose.

For example, in a moment of uncertainty and adversity, Participant B provided reassurance and encouragement to her friend:

I had a friend going to court, and she was so nervous. She did not know what to do. Then I helped her by telling her that when she arrives at court, she must speak the truth and face the challenges. This also motivated me to never give in life as I was struggling at school.

In this instance, Participant B’s empathy not only empowered her friend but also strengthened her own resilience, as supporting someone in crisis can cultivate a sense of agency and competence in the helper.

Participant F showed kindness by extending a helping hand to a friend in need. She said, “One of my friends did not have any food, and I was willing to also pack a lunch box for her and that made her very happy. For a first time in life, I felt needed and valuable to others”. In that way, Participant F created a supportive environment that fostered her resilience through connection and mutual care.

Furthermore, participant A encouraged a friend facing abuse to stand up for herself:

I had a friend whose stepfather was abusing her, and then she was afraid to tell her grandmother. I then told her that she should be standing up for herself, tell her grandmother and put an end to this. She then told her grandmother, and they reported the stepfather. I also learned from what my friend faced to also stand up for myself and face my challenges.

By advocating for her friend’s safety, Participant A not only contributed to her friend’s empowerment but also affirmed her own resilience by actively confronting the challenges posed by difficult situations. This sense of empathy and care serves as a protective factor, providing children who have been sexually abused with a renewed sense of purpose and fulfilment.

Based on the above quotations, participants were empathetic, supportive and caring towards other people. This sense of empathy and care is a protective factor, offering children who have been sexually abused a renewed sense of purpose and fulfilment. By assisting their friends to stand up and do what was needed, they confirmed that they too had learned from their experiences, which enabled them to assist others. Some participants mentioned that their experiences made them more resilient toward other problems life had brought their way.

#### Interests

Participants’ resilience was evident through engagement in activities like dancing, writing, singing, and celebrating birthdays. Their interest in these activities reflected a common human inclination to find meaning through creative outlets, expressing themselves, and enhancing their well-being. These interests included reading books, writing stories, dancing, and celebrating a friend’s birthday together.

Participant E described the pastimes she enjoyed. She said, “Dancing in my spare time. Reading novels and writing motivates and makes me calm when I am sad”. Participant E added, “Reading books, writing and drawing makes me happy”. Participant C explained why writing was important for her. She said, “I write down my feelings so that I can see how I have dealt with it. This helps me never to give up in life”. Through these creative expressions, Participant E cultivated resilience by channelling her emotions into productive activities, thereby enhancing her well-being. Participant F highlighted another interest—the tradition in their house dorm of celebrating birthdays collectively. They engaged in shared activities to honour the person’s birthday. Participant F said, “We will do activities together where we will all partake in someone’s birthday party, making it fun for everyone. This makes me feel supported and a sense of belonging”. The practice of celebrating birthdays as a group was meaningful to them and contributed to the development of togetherness. This practice not only provided her with insight into her emotional landscape but also served as a resilience-building strategy by promoting self-reflection and personal growth.

Moreover, the participants collectively expressed their passion for other activities, ranging from sports and academic endeavours to music and creative pursuits. This shared enthusiasm fostered a sense of camaraderie among them, creating a supportive and inclusive environment that further enhanced their collective identity. Participant C said,

I always used not to do sports at school. However, I really enjoy being part of sports because it helps me connect with others. Right now, I’m getting back into netball, and it’s becoming a favourite of mine. I also listen to music all day, write lyrics for new songs and then give them to someone to read. These activities help me cope with challenges in life.

Participant B said, “I have a passion for dancing, it makes me feel happy, and I enjoy working with children. I would be interested in becoming a social worker one day so I can help children like me”. Participant F expressed her interest in sports activities: “I really like sports such as athletics– long distance and hurdles. This helps me get rid of problems that make me feel sad”.

As evidenced by the narratives above, participants’ engagement in activities such as sports, listening to or creating music, reading books, writing, and dancing enhanced their personal growth, introspection, and self-expression, contributing to their overall well-being and resilience.

#### Bravery

This refers to the individual ability to face one’s fears and deal with trauma. This notion came to light through the insights provided by the participants. Participant C highlighted the invaluable lesson of cultivating courage: She said, “I learned that life is unpredictable. It sometimes surprises you, and you don’t have to let what happened in the past affect your present and the future. So, you just must move on. That’s what I taught myself”. By embracing the unpredictability of life, Participant C demonstrated resilience. Her proactive approach to her challenges allowed her to move forward despite past traumas.

Participant F shared a personal experience of bravery:

Yes, I had to be brave, and I felt that God gave me the strength to be brave. I stood up against a bully and walked away to talk to a teacher– and asked her to please help me or else I would fight with this person, as she would hit me or bring my mother into the fight. I learned that if someone fights me, I shouldn’t fight back because then it is me doing the same thing as that other person. Instead, I will go to a teacher and ask for assistance.

Participant F demonstrated resilience through bravery, choosing a constructive approach to conflict instead of resorting to violence.

Participant E concurred and gave her own example of courage:

I had to be brave one day, and the social worker helped me after I explained it to her. I learned from this experience that one should always feel comfortable; if you don’t feel comfortable, you need to tell an adult who you can trust. The thing that gives me hope is always doing what is right and not being afraid of other people.

Participant D added, “I must not allow others to make me feel uncomfortable. I’ve learned that I must fight for myself”. Participant A shared another aspect of bravery when she explained how she faced the situation when she had to go to the children’s home. She said, “I had to be brave coming to the children’s home because I didn’t want to leave my house. I never thought that I would live in a place like this but I am now happy and doing well in this place”.

Confronting fears and embracing bravery aids in cultivating a resilient self as portrayed by the participants of this study, even in the aftermath of adversity.

#### Vision

A vision acts as a guide for individuals’ aspirations and desires, shaping their ambitions and the person they strive to be. In the wake of trauma, individuals can harness this vision to navigate their path towards resilience. By envisioning a future that resonates with their values and aspirations, participants of this study embarked on a journey of healing and strength.

Participant B described the power of education. She said, “Education gives me hope for the future. I want to become a lawyer or a social worker so I can change the lives of children like me”. In a reflective moment, participant D also expressed her aspirations for the future. She said, “When I grow up, I aspire to become either a social worker or a songwriter. Being a social worker will allow me to protect children from bad things”. Such aspirations highlight how positive aspirations can be a source of resilience, propelling individuals forward despite past challenges. Participant F reflected on a hopeful future: “The things that I want to become one day– are something I can look forward to, especially, becoming a famous singer”. Participant C shared insights about her goals. She said, “I might not always show my talents, but I am the kind of person who enjoys rapping and creating my own music to express my emotions, especially when I can share it with other people in future”. Participant A affirmed, “No matter what challenges I face, I believe I will find a way through. Everything is possible and I will attain my goals of being a businesswoman”.

The abovementioned quotes showed that as children work towards actualising their vision, resilience takes root, helping them transform their pain into a powerful force for growth. Even after facing unimaginable adversity, such as sexual abuse, they can emerge as empowered individuals with a renewed sense of purpose, illustrating the remarkable capacity of the human spirit to rebound, be resilient and thrive.

### Relational Factors

Relational factors refer to interpersonal connections, specifically, examining how dependable social and contextual support from family and friends influences an individual’s ability to navigate emotional and physical adversities. Relational factors help to cultivate resilience, underscoring the profound impact of connections and support networks on an individual’s ability to overcome hardship.

Within the second theme, “Relational Factors”, subthemes intertwine: (1) Supportive social connections (2) Caregivers’ support, and (3) Teamwork and collaboration.

#### Supportive social connections

In this study, supportive social connections played an important role in building resilience for participants. These connections were lifelines amidst the storm of trauma. Participants in this study reported reciprocal supportive social connections. Participants supported their friends, and they were in turn supported by their friends. Participant E underlined her dedication to aiding friends: “I will not let my friends down. I encourage them to continue to persevere, and if they fail, I encourage them to try again”. This commitment to supporting others reflects a key aspect of resilience—recognising that together, individuals can withstand challenges and encourage perseverance in each other. Participant E shared how supporting her friend in a challenging situation played a significant role in building her resilience. She said, “I supported my friend who was dealing with bullying. I informed the teacher and sought help from an adult, this made me believe in myself.” She emphasised that taking action not only helped her friend but also strengthened her own confidence and sense of responsibility. Her proactive approach demonstrated resilience through advocacy and the willingness to seek help, highlighting how standing up for others contributed to her personal growth and emotional strength. The participants shared stories of helping each other during difficult times and providing each other with emotional support.

During challenging moments, friends extended their support to the participants by offering essential resources, even when they were not needed. As participant F commented: “Friends from other schools will ask me if I need something like toiletries or any household items that I use daily. Even though I said, ‘no thank you’, they still decided to bring”. She explained how these acts of kindness provided not only practical help but also emotional reassurance, reinforcing her sense of community and belonging. Their unwavering support strengthened her ability to navigate difficulties with confidence.

Participant B added:

My roommate always supports me when I am going through a lot. When my case is nearby, and I need to go to court, she helps me to reduce my stress by saying I need to pray, listen to others, and show respect to my elders.

The participants thus highlighted the critical role of social connections in nurturing resilience.

The support from Participant B’s roommate demonstrated the protective effect of social connections, showing that having someone to lean on during stressful times can enhance one’s ability to cope with adversity.

#### Relationship with caregivers

This study explored how children who had faced sexual abuse derived resilience from their relationships with caregivers, including social workers and housemothers. The study highlighted caregivers as the primary support system, providing essential resources and fostering a secure environment with trust and open communication. This subtheme emphasised the role of caregivers’ support in nurturing resilience among participants in the face of adversity. To illustrate this theme, participant E said, “I am grateful for my social worker at the children’s home as they will make me feel safe and loved by giving me a space to talk freely about my problems or something that bothers me”. This gratitude reflects how a safe and supportive relationship with a caregiver can foster resilience by providing emotional stability during turbulent times. Participant F echoed this sentiment.

The housemother who is at the children’s home, assisted me in challenges. Like the

one day, I was sad and felt different emotions. Then she assisted me in doing an activity like building an emotion monster. We had to take a picture, and I wrote that I feel safe in the housemother’s heart and can truly talk to her about problems because then she will be able to support me, and we can always make a plan.

Her experience illustrates how emotional support not only provided her with comfort but also reinforced her ability to express and process emotions in a healthy way, strengthening her resilience.

Participant B described the sense of security and affection she experienced. She said, “The caregivers make me feel safe and loved by giving me space to talk about my problems, which wasn’t the case at my parent’s house”. These narratives emphasise the importance of caregivers in fostering resilience through a sense of safety while providing a space for open communication and healing.

#### Teamwork and collaboration

Teamwork and collaboration play a pivotal role in constructing participants’ resilience. The participants collaboratively worked in teams, leveraging each other’s strengths to actively tackle tasks and apply their collective skills to accomplish the required assignments. Participant B shed light on the way she worked with others. She said, “I came with my ideas, and she came with her ideas. Then we combined them. Then yeah, the task was done”. Similarly, participant E stated, “Yes, we helped each other by dividing the work to everyone so that each participant does a part of the assignment such as, one will write, the others will do research or compile the document”. Participant F shared, “We sat together and agreed on what everyone should do and how we can bring the project together. One will give the idea while others write or do the research”. Sharing responsibilities contributed to the participants’ journey towards resilience amid challenging tasks.

### Contextual Factors

The third theme, “Contextual Factors”, delves into the broader environmental contribution to participants’ resilience.

There were three significant subthemes that emerged under the third theme: (1) Role models (2) Religion and spirituality and (3) Support in educational settings.

#### Role models

Having role models enabled participants’ resilience. Role models embody qualities of affection and inspiration, being who they are, giving advice and hope. Participant E stated, “My role model is one of the ladies working at the children’s home. She is a role model because she shows affection towards other people. She always listens to me and gives guidance”.

Participants identified role models who exemplify resilience, drawing inspiration from their love of music and the art of writing lyrics. For example, participant F specified, “There is a lot that I see in a role model that keeps me going, because I love their music, and I would like to sing my songs as they do one day”. This connection to role models showcases how inspiration can fuel aspirations, enabling participants to envision a brighter future despite their current adversities. Participant B shared her personal aspirations: “I have a passion for dancing, and I enjoy working with children. I would be interested to become a social worker one day as I see how social workers have helped me”. This aspiration indicates the positive impact of role models that may help them contribute to the well-being of others, enhancing their resilience through purpose.

Participant C added,

Yeah, my role model is a rapper. He’s the person, and I always watch his music videos on television. And then I started downloading his songs, and I started getting interested in hip hop because I was listening to his songs. And then I started also researching about his background, his life and stuff. Then I also got to know that he also grew up without his mother and his father like me. And then, yeah, it was only him and his siblings. And then he grew up, and he’s now known as South Africa’s biggest rapper. Yes, he is the person who made me interested in music and he motivates me to follow my dreams even though I don’t have parents.

Therefore, positive role models provided hope for the future, which enabled the participants to bounce back despite their adversities. The history and success of their role models motivated them to pursue their aspirations.

#### Religion and spirituality

In this study, religion and spirituality were found to be protective factors for children who had been sexually abused. All participants highlighted their engagement in spiritual or religious pursuits, which they found fulfilling and significant.

Participant E shared that church activities gave her strength. She said, “Church activities help me to cope with challenges because I can read the Bible and then escape from reality”. This engagement with religious practices provides a source of strength and comfort, underscoring the role of spirituality in building resilience. Participant A enjoyed their religious practices, “Well, we go to church every Sunday. And like this coming Friday, we will have something for our youth to give us hope in life”. Participant E found it valuable to discuss personal challenges with others, remarking, “It helps to talk about my problems to someone who can help me from a more religious perspective”. This indicates that shared spiritual experiences can provide a supportive network, enhancing resilience by encouraging individuals to express their feelings and seek guidance. Participant C added, “I believe in God, and I fear nothing as He is with me always”. This profound sense of faith and assurance illustrates how spirituality can serve as a powerful anchor, providing resilience amid life’s challenges.

These narratives revealed that religion and spirituality are significant sources of resilience. They provided all the participants with a sense of purpose and inner strength. Reading the Bible, attending church services, socialising with other churchgoers, and spiritual guidance offered them an alternative to life’s adversities.

#### Support from schoolteachers

Support in school settings was found to enhance resilience among the participants of this study. They could work through their personal challenges in supportive and empathetic environments with the assistance of teachers. Participant F expressed her gratitude towards the schoolteachers. She said, “I am thankful for the teacher or person who protects me or helps me when I am in trouble”. Participant B appreciated the encouragement to raise her standards: “All the teachers at school support me by putting more pressure on me and saying you must put more effort in your studies so that you can make your future bright”. Educators play a vital role in fostering resilience by pushing students to achieve their goals and providing a sense of accountability. Participant C confirmed, “School supports me through the kind of teachers that we have. Even though most of them are strict, they help me stay focused on school”.

This contextual network of support at school played a protective role by mitigating the challenges the children faced. This enabled them to cope with the difficulties they faced.

## Discussion

This study sought to document the resilience of children who had experienced sexual abuse. The main purpose of the study was to investigate factors that aided them in developing resilience despite such adversity. The main finding of this study was that the participants’ resilience was due to complex combinations of individual, relational and contextual factors. These findings provided significant insights into the complexity of resilience, expanding on existing literature through a qualitative study that introduced new protective factors while reaffirming existing social-ecological resilience-enabling resources.

The study identified several individual factors that played a critical role in enabling the resilience of the participants. Yoleri (2020) defined individual factors as the inherent capacity of a person to swiftly adapt to changing conditions, address challenges expediently, and generate a broader spectrum of solutions to problems. This expanded perspective aligns with the broader concept of resilience, emphasising the adaptive, problem-solving capacity of individual factors within the resilience framework. Within the social-ecological theoretical framework that underpins this study, Ungar (2013) stated that resilient individuals maintain a belief in their control over their destiny, despite challenges. This suggests that resilience-enabling traits interact and reciprocally reinforce each other, underscoring the interconnected nature of resilience-enabling factors within the individual. In this specific study it was found that individual factors included the ability to help others, nurturing personal interests, displaying bravery, and a vision for the future. These traits align with previous research on resilience (Burns et al., 2013; Mawila, 2023; Theron, 2010; Van Rensburg et al., 2015), which pinpointed qualities such as empathy, conscientiousness, self-regulation, optimism, flexibility, and assertiveness as resilience-enhancing attributes.

The emergence of altruism—helping others—as a protective factor is particularly notable. This research provided evidence through participants’ verbatim responses that helping others can foster self-worth and emotional strength, serving as a powerful resilience factor in the aftermath of sexual abuse. This underscores the importance of communal care and empathy as central to the resilience-building process in children who have experienced sexual abuse.

In this study, engaging in creative outlets — such as dancing, writing, singing, and celebrating milestones like birthdays — was found to be instrumental in anchoring participants’ resilience. This aligns with Myburgh (2015), who emphasises the human tendency to find meaning through artistic expression, which contributes to psychological well-being. Prior studies (Dimopoulos et al., 2016; Omanov, 2021) also demonstrated how creative activities promote cultural identity and personal growth. Notably, this study expands on previous findings by specifically underscoring the importance of these activities in helping children who have been sexually abused. Another prominent individual factor in this study was bravery, which participants linked to their ability to confront difficult situations and cope resiliently. Lodi et al. (2022) explain that bravery enables individuals to face fears and hardships. In studies of sexual abuse, Denney (2021) also suggests that bravery involves moral integrity in confronting difficult situations. Thus, this study adds to the understanding of bravery as a personal strength in anchoring resilience in the context of sexual abuse.

Finally, having a vision for the future emerged as a significant contributor to resilience. Participants demonstrated how the ability to imagine a positive future helped them navigate adversities and remain resilient. This finding mirrors that of Richter et al. (2018), who indicate that intentional aspirations and positive future outlooks play pivotal roles in fostering resilience. Forward-looking beliefs and goals played a pivotal role in maintaining a positive perspective and adapting to challenges, aligning with studies on the resilience of sexually abused individuals (Gower et al., 2020; Phasha, 2010). The theme of “having a vision” was consistent among participants, acting as a wellspring of hope and motivation (Domhardt et al., 2015). Evidently, having a vision of the future is a protective factor for children who have been sexually abuse, however, it has not been fully explored in the literature.

Another socio-ecological factor reported by the participants was the connection with significant people. This connection falls within the relational factors that foster resilience. Participants expressed the significant role of family relationships, particularly the support of caregivers, housemothers, and social workers. The positive involvement of caregivers and housemothers in creating a stable and supportive environment for participants reflects how relational bonds serve as foundational pillars of resilience. The findings of this study align with Gower et al. (2020), who underscored the critical role of caregivers in fostering resilience. Despite the significant emotional toll of sexual abuse, peer relationships were found to be crucial to the resilience of participants. Olekalns et al. (2020) argue that positive peer relationships are essential to resilience, particularly in challenging environments. Haffejee and Theron (2017) additionally highlighted research by Aspelmeier et al. (2007), which found that these bonds act as social buffers, helping children navigate their trauma by offering empathy, companionship, and solidarity.

Ungar (2012) highlighted resilience as a complex construct shaped by contextual and cultural factors that influence an individual’s development, with particular emphasis on social and environmental contributions to personal processes and outcomes (Vaughn & Dejonckheere, 2021). In his framework, Ungar (2011) argued that a comprehensive understanding of resilience requires attention to social ecological supports and the quality of assistance these environments can provide, particularly for at-risk children. Consistent with Ungar’s theory, contextual factors like positive role models, teacher support, and religious or spiritual beliefs were found to play an essential role in fostering resilience. The presence of positive role models — teachers, caregivers, and community members — proved pivotal, offering guidance and inspiration that promoted resilience by enhancing self-worth and self-efficacy (Masten, 2014; Valero et al., 2019). These findings, which underscore the importance of social supports, add new insights into the role of role models in resilience-building, particularly for children who have experienced sexual abuse.

Spirituality and religion also emerged as significant protective factors, with participants describing how faith practices such as attending church and reading religious texts helped them cope with their adversities. This aligns with Mokgopha (2019), who asserts that spirituality promotes positive interpersonal relationships and emotional strength. Furthermore, research by Young et al. (2007) and Gower et al. (2020) links spirituality with resilience in the face of adversity. Lastly, school support, particularly from teachers, was another key contextual factor that enhanced the participants’ resilience. Teachers provided emotional and psychological support, championing their resilience during difficult times. Malindi and Machenjedze (2012) emphasise the importance of school environments in nurturing resilience. Allen et al. (2008) and Bogar and Hulse-Killacky (2006) also affirm the crucial role that teacher-student relationships play in fostering resilience.

Ungar’s social-ecological theory of resilience provides a comprehensive framework that illustrates how various systems interact to shape beliefs, build connections, and influence broader societal contexts. This study, based on a qualitative investigation within a South African context, contributed to understanding resilience in children, particularly those who have experienced sexual abuse in children’s homes. By integrating individual, relational, and contextual factors, Ungar’s theory (2015) illustrates how children navigate adversity and leverage resources within their environments. In line with the Ubuntu principles of togetherness, different stakeholders (such as caregivers, housemothers, friends and teachers) in this study were co-responsible for facilitating positive adjustment in children who have been sexually abused. van Breda (2018) and Theron and Phasha (2015) reinforce Ubuntu’s description of people’s interdependence or interconnectedness as foundational to personhood and a cornerstone of resilience. This study underscores the reciprocal nature of person-environment interactions within the social-ecological framework. In conclusion, the findings of this current study contribute new insights into the application of Ungar’s social-ecological theory, particularly its relevance in enabling the resilience of children in South African children’s homes who have experienced sexual abuse.

### Limitations

This qualitative study aimed to understand the resilience of children who faced sexual abuse. While the findings provided valuable insights, it is important to acknowledge certain limitations. One limitation relates to the semi-structured interviews, where the questions chosen by the researcher could have influenced which protective factors were mentioned by the participants. Additionally, the reliance on interviews alone may not have captured the full spectrum of experiences, although they were sufficient for gathering phenomenological information. The absence of other methods of data collection, such as focus groups, was due to ethical considerations regarding the confidentiality of participants, given the sensitive nature of the study. Art-based data collection methods could be explored in future studies with children who have been sexually abused. Furthermore, even though the research in this study was conceptualised comprehensively, the use of a priori coding and the construction of interview questions based on Ungar’s framework may have influenced the emergence of themes; findings might have been shaped by the predetermined coding structure rather than arising organically from the data. Future research could explore more inductive approaches to allow for a broader range of resilience factors to surface, ensuring that themes emerge more naturally from participants’ lived experiences.

This study focused exclusively on female participants, resulting in a restricted sample size that may limit the generalisability of the findings. A critical constraint also emerged regarding the applicability of the study’s findings beyond the two children’s homes in South Africa. It is imperative to exercise caution when extrapolating the outcomes to other children’s homes. The sensitive nature of the subject matter necessitates caution in generalising findings to broader populations or making gender-specific assertions. However, the study mitigated this limitation by diligently collecting detailed information from each participant, prioritising quality over quantity. Future studies could enhance the richness of the data by incorporating art-based techniques such as drawings, collages, and pictures. The qualitative approach enabled an in-depth exploration of the lived experiences of the participants, fostering a deeper understanding of individual perspectives (Tenny et al., 2022).

### Implications and Future Research

The findings of this study underscored the importance of viewing the resilience of children who have been sexually abused from multiple perspectives. Resilience is not solely an individual trait but is shaped and influenced by the protective factors in the collective efforts of people and the environments surrounding an individual. This collaborative approach enriches and expands the understanding of resilience by emphasising the dynamic interactions between different systems and individuals in promoting positive outcomes after traumatic experiences. By advocating for collaboration among various stakeholders, the study reinforced the importance of multiple levels of support in fostering resilience in individuals who have experienced sexual abuse.

In addition, the study provided valuable insights, which could be considered by mental health practitioners in therapeutic processes, educational programmes, and support services aiming to build the resilience of children who have been sexually abused. We recommend implementing resilience-building workshops tailored for children who have experienced sexual abuse. These workshops could use strategies such as expressive arts activities, maintaining relationships with others and utilising accessible contextual resources. Moreover, the researchers recommend that children’s homes strengthen communication channels to create a secure environment for children who have faced sexual abuse. This would encourage them to express their emotions without fear of judgement and would enhance their resilience. Children’s homes are urged to collaborate with their own stakeholders and with external support networks, including connections with counsellors and therapists. This is the cornerstone of a comprehensive multidisciplinary approach to resilience.

Given the dearth of knowledge on the factors that enable the resilience of children who have faced sexual abuse, this study contributed to filling this knowledge gap. Similar studies should be conducted in different contexts. These studies could expand the scope of this study and include various children’s homes nationwide, with both female and male participants. In conclusion, the findings demonstrated that children who have been sexually abused can show impressive resilience despite the adversities they face. Their resilience depends on their personal attributes, the relationships they have with other people and available resources within their context. Thus, this study confirmed that resilience is based on a combination of multiple factors.

## Data Availability

Anonymous data of the study is available upon reasonable request.
